# Microwave ablation followed by cTACE in 5-cm HCC lesions: does a single-session approach affect liver function?

**DOI:** 10.1007/s11547-024-01842-7

**Published:** 2024-07-03

**Authors:** Chiara Floridi, Laura Maria Cacioppa, Nicolò Rossini, Marco Macchini, Alessandra Bruno, Andrea Agostini, Valeria Consoli, Riccardo Inchingolo, Fabrizio Acquafredda, Daniele Nicolini, Laura Schiadà, Gianluca Svegliati Baroni, Roberto Candelari

**Affiliations:** 1 Division of Interventional Radiology, Department of Radiological Sciences, University Politecnica Delle Marche, 60126 Ancona, Italy; 2 Division of Interventional Radiology, Department of Clinical, Special and Dental Sciences, University Politecnica Delle Marche, 60126 Ancona, Italy; 3 Division of Radiology, Department of Radiological Sciences, University Hospital “Azienda Ospedaliero Universitaria Delle Marche”, 60126 Ancona, Italy; 4 Interventional Radiology Unit, “F. Miulli” Regional General Hospital, 70021 Acquaviva Delle Fonti, Bari, Italy; 5 Hepato-Pancreato-Biliary and Transplant Surgery, Department of Experimental and Clinical Medicine, Polytechnic University of Marche, 60126 Ancona, Italy; 6 “Transplant and Hepatic Damage” Unit, University Politecnica Delle Marche, 60126 Ancona, Italy

**Keywords:** Hepatocellular carcinoma, Liver disease, Therapeutic chemoembolization, Interventional oncology, Ablation, Microwaves

## Abstract

**Purpose:**

Microwave ablation (MWA) and conventional transarterial chemoembolization (cTACE) are locoregional treatments commonly performed in very early, early and intermediate stages of hepatocellular carcinoma (HCC). Despite combined locoregional approaches have shown encouraging results in obtaining complete tumor necrosis, their application in a single session is poorly described.

Our aim was to evaluate the safety and efficacy of single-session MWA and cTACE treatment in 5-cm HCCs and its influence on liver function.

**Materials and methods:**

All 5-cm HCCs treated by MWA and cTACE performed in a single-session in our Interventional Radiology unit between January 2020 and December 2022 were retrospectively recorded and analyzed. Patients with poor or missing pre- and post-treatment imaging were excluded. Technical success, clinical success, and complications rate were examined as primary endpoints. Pre- and post-treatment liver function laboratory parameters were also evaluated.

**Results:**

A total of 15 lesions (mean lesion diameter, 5.0 ± 1.4 cm) in 15 patients (11 men; mean age, 67.1 ± 8.9 years) were retrospectively evaluated. Technical and clinical success were 100% and 73%, respectively. Four (27%) cases of partial response and no cases of progressive or stable disease were recorded. AST and ALT values have found to be significantly higher in post-treatment laboratory tests. No other significant differences between pre- and post-treatment laboratory values were registered. AST and ALT pre- and post-treatment higher differences (ΔAST and ΔALT) were significantly associated with a lower clinical success rate.

**Conclusion:**

MWA and cTACE single-session approach is safe and effective for 5-cm HCCs, without significant liver function impairment. A post-treatment increase in AST and ALT values may be a predictor for clinical failure.

## Introduction

Hepatocellular carcinoma (HCC) is the most common type of primary liver cancer and a leading cause of cancer-related death [1, 2]. Although cirrhosis is a well-known principal risk factor for HCC, several minor co-factors with synergic effect exist [3]. At-risk patients are closely monitored with abdominal ultrasound, alpha-fetoprotein (AFP) biomarker and liver function laboratory tests, whereas CT and MRI are not performed routinely [4–8]. Over the past decade, HCC incidence and mortality have remained stable due to the active surveillance programs, the multidisciplinary approach and the improvements of locoregional and systemic treatments [4, 9–11].

Locoregional minimally invasive procedures, such as microwave ablation (MWA) and transarterial chemoembolization (TACE), are, respectively, indicated for very early/early stage HCC and unresectable intermediate-stage HCC, according to Barcelona Clinic Liver Cancer staging [12, 13]. In addition, multimodal and combined locoregional approaches have been developed with the aim of reaching the best match for every clinical scenario [14–20]. The combination of TACE with ablative therapies has been proven to increase treatment efficacy, mostly preventing incomplete peripheral tumor necrosis and especially in largest tumors sized 3–7 cm, without adjuntive risks [18–20].

The rationale of the combined application of these two strategies stems from the variable responses of large HCCs to TACE, due to the development of hypoxia and consequent angiogenesis, often hesitating in tumor progression or recurrence. This phenomenon could be mitigated by MWA, usually not performed in large, intermediate-stage HCCs but efficiently applicable in viable tumors after TACE [21].

Despite the known advantages of combined (early or late) sequential MWA and TACE, its application in single session has been poorly described in literature due to the assumption that a shorter time interval between the MWA and TACE deteriorates patient liver function [21, 22]. Our study aims to evaluate safety, technical and clinical success, and the effect on liver function of single-step MWA and conventional TACE (cTACE) combined procedures.

## Materials and methods

### Study design and patient population

The study was approved by the Internal Review Board (IRB) and was conducted in conformity to the ethics guidelines of the 1990 Declaration of Helsinki and its amendments. All patients provided an informed written consent to the procedures.

This single-center retrospective study analyzed all the consecutive patients referred to our Interventional Radiology unit for unresectable HCCs with main diameter ≤ 6-cm from January 2020 to December 2022. All the unresectable HCCs ≤ 5-cm treated by MWA followed by cTACE in a single-session approach, with available pre- and post-treatment Contrast Enhanced Computed Tomography (CECT) performed in our center, were included.

Files and images were extracted from RIS (Radiology Information) and PACS (Picture Archiving and Communication Systems—GE Medical System, Milwaukee) of the hospital. The searched key words were “hepatic chemoembolization” and “hepatic ablation”. Demographic, clinical and laboratory data were obtained from digital medical records.

Treatment decision of all the included cases were based on a multidisciplinary consensus obtained during dedicated tumor board meetings attended by a dedicated oncologic radiologist, an interventional radiologist, an oncologist, an hepatologist and a liver transplant surgeon. All patients were deemed unfit for surgery due to lesion characteristics, patient’s comorbidities, or refusal.

Inclusion criteria were Child–Pugh score A, liver cirrhosis, single unresectable ≤ 6-cm HCC or < 3 multinodular HCC and a target lesion ≤ 6-cm, absence of extrahepatic disease on CT imaging.

**Fig. 1:**
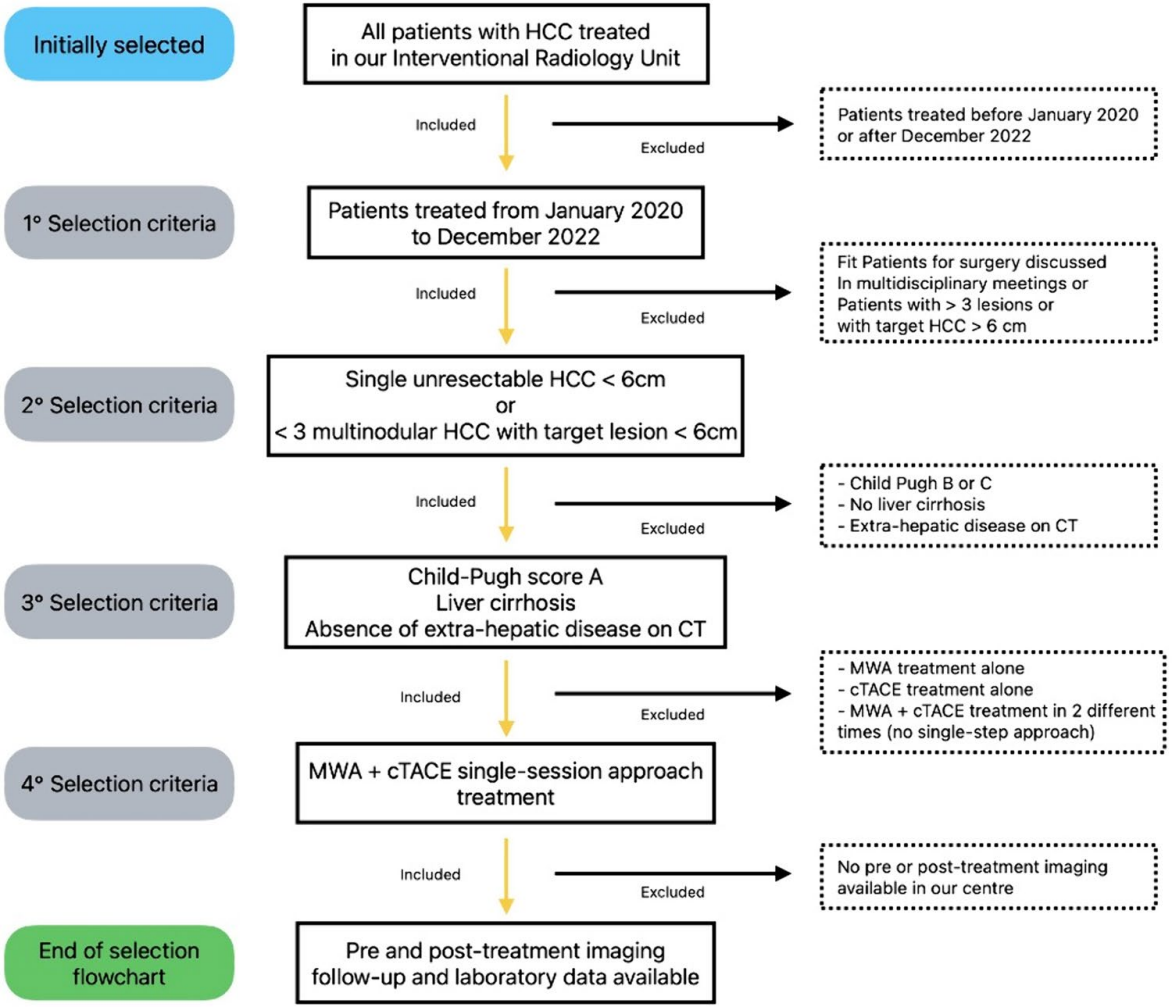
Flowchart explaining patients selection procedure in detail

Exclusion criteria were age < 18 years, known allergy to iodinated contrast medium, impaired platelet count, and coagulation parameters (INR > 1.5; PLT < 45,000/μL) (Fig. 1).

### Clinical and laboratory characteristics

Patient general characteristics (age and sex), principal comorbidities, previous surgical and locoregional therapies, HCC nodules number, size (main diameter) and volume were assessed. Aspartate Transferase (AST), Alanine Aminotransferase (ALT), Gamma-Glutamyl Transferase (GGT), Alkaline Phosphatase (ALP), INR (International Normalized Ratio), aPPT (activated partial thromboplastin time), platelet (PLT) count (× 103/mmc), albumin (ALB); total, direct and indirect bilirubin values (Bil Tot, Bil Dir and Bil Ind, respectively), and Alpha-fetoprotein (AFP) levels measured within 24 h before and 24 h after a single-session locoregional procedure were evaluated. The difference between pre- and post-procedural values of each parameter was calculated and defined as Δ (delta). In addition, the variation of these values between pre- and post-treatment, reported as ΔAST, ΔALT, ΔGGT, ΔALP, ΔPLT, ΔALB, ΔBilDir, ΔBilInd, ΔBilTot and ΔAFP were evaluated and included in the statistical analysis. Laboratory values were also registered 7 days after the procedure.

### Diagnostic imaging

All cases had previously undergone thorax and abdomen CECT pretreatment study. One and three-month CECT follow-up imaging was available.

CT standard protocol consisted of basal unenhanced phase followed by intravenous injection of contrast medium (1.5 ml/kg, Iopamiro®370, Bracco, Milan, Italy) and dedicated triple-phase liver protocol (arterial phase, portal venous phase and delayed phase acquisition). All CT scans were realized with 64-Multi Detector CT (Lightspeed VCT, GE Healthcare, USA) or with 256-slice Dual Scan CT (SOMATOM Force, Siemens Healthineers, Forchheim, Germany). Furthermore, all the patients had previously being submitted to a liver Ultrasound (US; RS80a, Samsung) to evaluate the feasibility of US guidance as an aid to MW-probe positioning in addition to CBCT guidance.

### Cone beam CT imaging

CBCT imaging was obtained during end-expiration apnea and using two different angiographic units both equipped with a digital monoplane C-arm Cone beam CT (Artis zee, Siemens Healthcare GmbH, Erlangen, Germany) and (Azurion, Philips Medical Systems, Amsterdam, Netherlands).

**Fig. 2:**
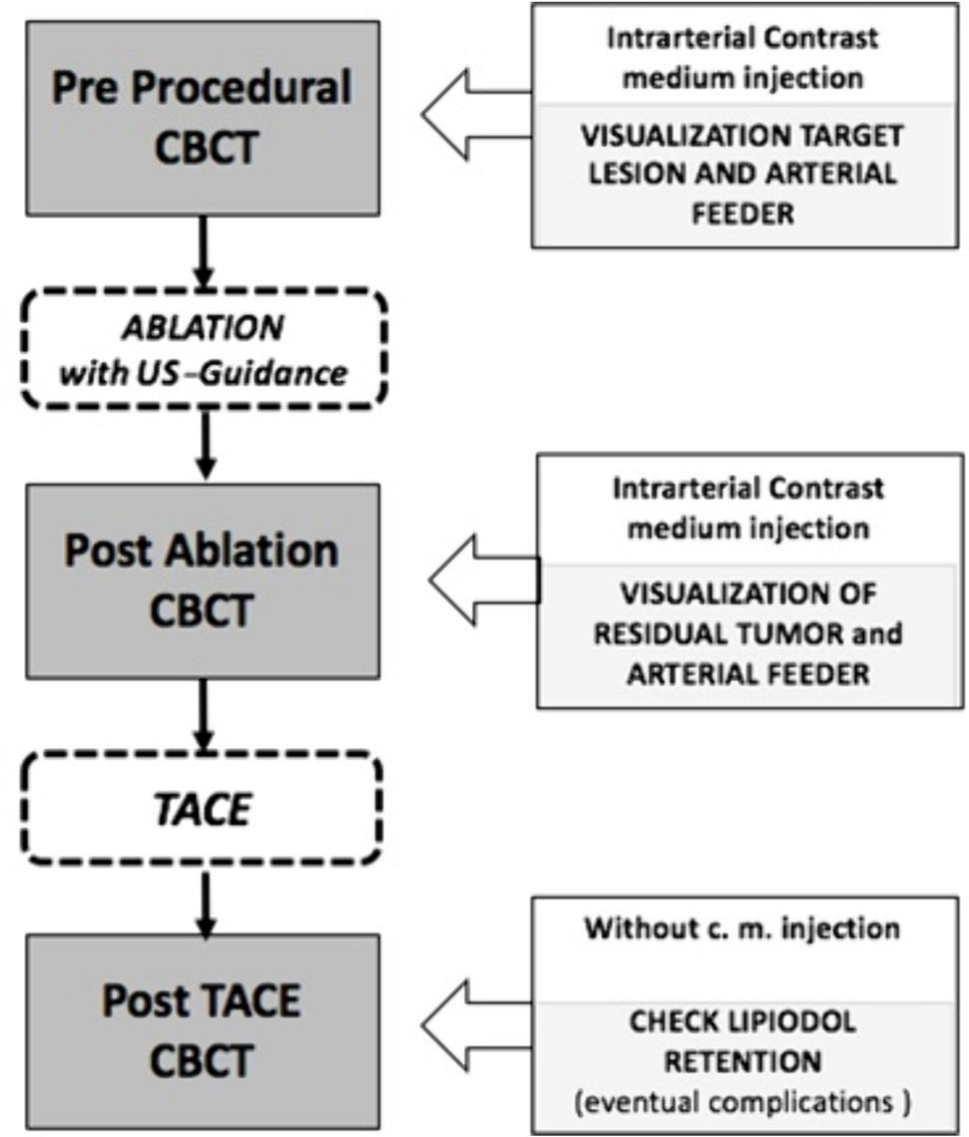
Diagram showing methodology of Cone beam CT (CBCT) intraprocedural protocol consisting of three acquisitions

The department’s standard protocol, summarized in Fig. 2, consisted of three acquisitions including:

1. Pretreatment dual-phase contrast enhanced CBCT (ceCBCT), obtained by intra-arterial contrast medium injection through the diagnostic catheter with the aim of confirming the size and location of the target lesion, identifying any additional nodule undetected at preliminary CT, mapping the feeding arteries, and excluding potential arteriovenous shunts;
2. Post-MWA-ceCBCT, obtained by intra-arterial contrast medium injection through the diagnostic catheter to evaluate the ablated area, to visualize the residual tumor where cTACE needs to be performed;
3. Post-cTACE-CBCT obtained without contrast medium injection to evaluate lipiodol retention and its distribution compared to the ablation area.

### Single-Session combined procedure

All the combined treatments were performed in two fully equipped angio-suites, in a single-session approach, with patient monitoring and anaesthesiologic assistance. MWA and subsequent cTACE procedures were performed by an interventional radiologist with at least 5 years of experience.

A right common femoral artery access was performed under local anaesthesia (10ml of Mepivacaine 2% solution), and a 4Fr introducer sheath was positioned. The main hepatic artery was catheterized using 4Fr catheters such as Cobra-2 and Simmons-1 (Cordis, Santa Clara, CA, USA), and a preliminary selective arteriography was performed. Pretreatment imaging was completed by a contrast enhanced CBCT protocol, as previously described.

Following conscious sedation, percutaneous US-guided MWA (Solero, Angiodynamics, Latham, NY, USA) was performed after placing the MW-probe tip in the optimal central position, according to the predetermined MWA scheme.

**Fig. 3:**
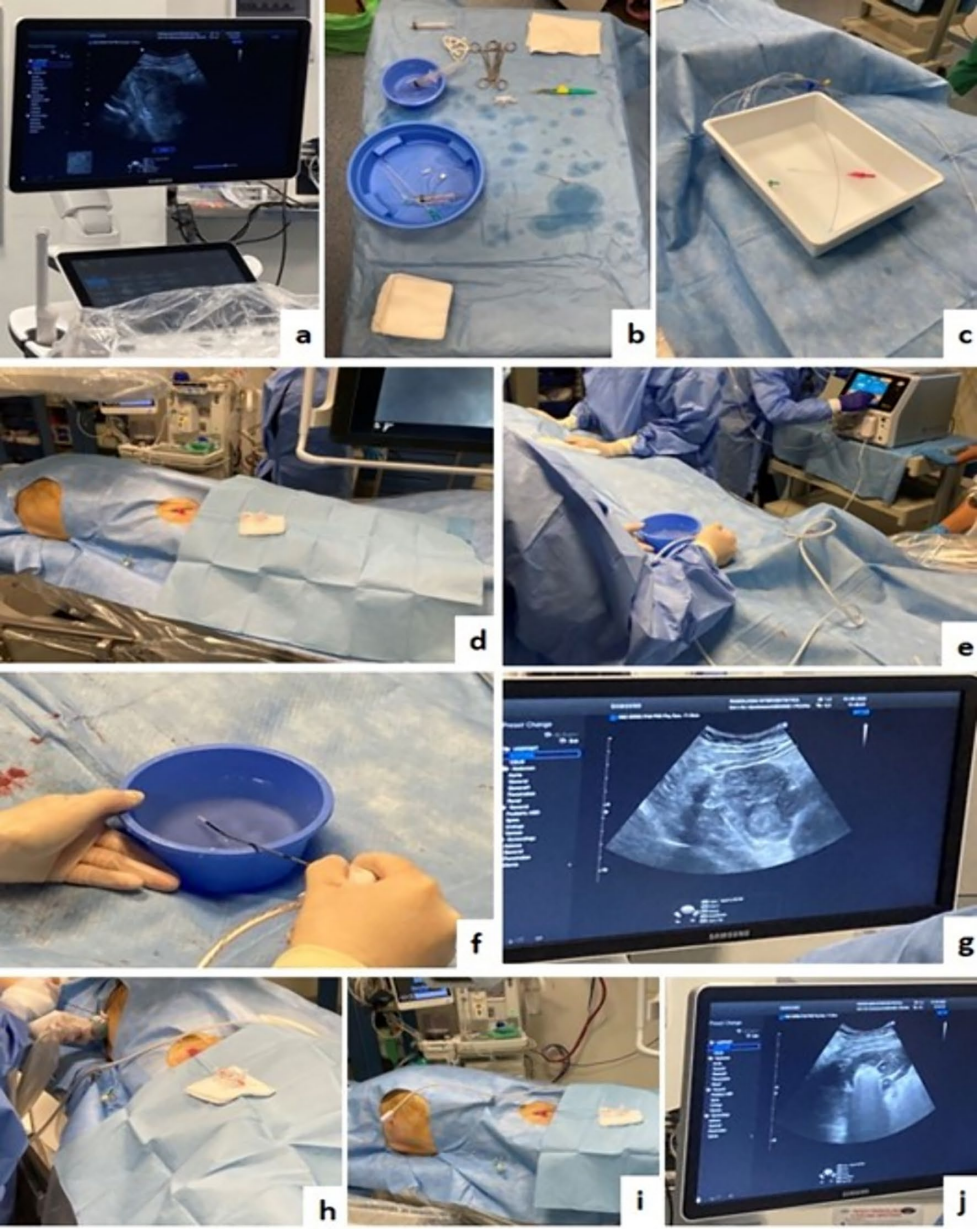
technical steps of combined single-session MWA + cTACE procedure. Preliminary liver US evaluation (**a**); interventional sterile table and 4Fr introducer sheath preparation (**b**–**c**); femoral access with positioning of 4Fr introducer sheath (**d**); MW-probe opening and testing (**e**–**f**); second ultrasound evaluation before MW-probe positioning (**g**); ultrasound-guided ablation (**h**); panoramic view of the two accesses, one percutaneous with the antenna-probe onsite, and one on the right groin for cTACE (**i**); ultrasound monitoring during ablation showing typical acoustic shadowing artifacts (**j**)

Once MWA was completed and the MW-probe was withdrawn, a post-MWA CBCT was performed and the patient underwent cTACE following catheterization of the tumor-feeding arteries using 2.4–2.7 Fr microcatheters (Progreat, Terumo Medical, Tokyo, Japan), as selectively as possible. An emulsion of 2–20 mL of Ethiodized oil (Lipiodol Ultra-Fluid; Laboratoire Andre Guerbet, Aulnay-sous-Bois, France) and 75 mg of doxorubicin hydrochloride was then administered with a dosage determined according to tumor size and patient’s liver function, until flow stasis was achieved. Consequently, Gelatin sponge was administered in “slurry” preparations (Spongostan®, Johnson & Johnson Medical NV, NJ, USA). Finally, post-cTACE-CBCT scan was performed, and the catheter was removed. Haemostasis of the puncture site was obtained by manual compression. The average time between the end of MWA and the beginning of drug injection was approximately 5 min. The main steps of the single-session approach are summarized in Fig. 3. No antibiotic prophylaxis was realized before and after the single-session combined treatment.

### Definitions and outcomes

Technical success was defined as a MWA followed by cTACE successfully performed in a single-session approach. Inadequate US window preventing safe percutaneous access for MWA, failure of arterial branch catheterization, and inefficient catheterization resulting in vessel damage were considered as cases of technical failure.

Clinical success was defined as the absence of residual HCC at 1- and 3-month CECT follow-up, defined as complete response (CR) according to mRECIST criteria [23]. Cases of stable disease (SD) or partial response (PR) were deemed clinical failures. Complications were evaluated based on the CIRSE classification system [24, 25].

The assessment of single-session MWA + cTACE impact on liver function was also evaluated as a secondary endpoint. This analysis was conducted in terms of post-procedural variation of the liver laboratory panel parameters listed above.

### Statistical analysis

Data were analyzed using GraphPad Prism version 9.1.1 (GraphPad Software, Boston, MA) statistical software. Counts and percentage were used to report nominal variables whereas means and standard deviations (95% confidence interval) were used for continuous variables.

The Shapiro Wilk Test was performed to determine the categorical variables as appropriate.

Student’s t test was used for continuous variables with normal distribution (Gaussian continuous variables) whereas for continuous variables with abnormal distribution (non-Gaussian continuous variables) and for ordinal variables, Mann–Whitney U test was used.

A *p* value < 0.05 was considered statistically significant. For each statistical comparison, confidence intervals (CI) and p values were reported.

## Results

A total of 15 HCCs in 15 patients (11 male; 71.4%) were treated. The mean age was 67.1 ± 8.9 years (range:51–85 years). Clinical comorbidities were present in 12/15 patients (86%).

The mean lesion diameter was 5 ± 1.4 cm. Four (27%) target lesions were localized in left liver lobe, and 11 (73%) in the right lobe. Demographics, clinical findings, and lesion characteristics are summarized in Table 1.

**Table 1:** Demographics, clinical and local disease characteristics of study population

	n (%)
*Demographics*	
Patients	15
Procedures	15
Age (years) (Mean ± SD)	67.1 ± 8.9
Age range (years)	(51–85)
Males	11 (73%)
Females	4 (27%)
*Clinical comorbidities (presence)*	
Yes	13 (87%)
No	2 (13%)
*Clinical comorbidities (type)*	
Hypertension	10 (66%)
Diabetes mellitus	2 (13%)
Cardiovascular disease	3 (20%)
Previous hepatic surgery	0 (0%)
Previous locoregional treatments	0 (0%)
*Cirrhosis*	
Yes	15 (100%)
No	0 (0%)
HCC nodules size (cm)	5.0 ± 1.4
*HCC site*	
Left lobe	4 (27%)
Right lobe	11 (73%)

Mean laboratory values of AST, ALT, GGT, ALP, BilDir, BilInd, BilTot, ALB, PLT and AFP as well as their variations between pre- and post-procedural values (Δ), and their values registered 7 days after procedures are analytically reported in Table 2.

**Table 2:** Laboratory characteristics of study population including pre-procedural values, post-procedural values and their modifications between pre and post-procedure (Δ). Average values at 7 days after procedures were also reported

	Pre-procedural	Post-procedural	Δ	7-day values	
Liver Injury indices	AST (U/L)	40.7	156.1	108.6	84.6
	ALT (U/L)	49.0	125.5	63.6	79.5
Cholestasis indices	GGT (U/L)	153.5	132.7	− 10.4	119.6
	ALP (U/L)	114.7	99.3	− 3.4	97.8
	Bil Dir (mg/dl)	0.32	0.64	0.3	0.52
	Bil Ind (mg/dl)	0.65	1.2	0.4	0.76
	Bil Tot (mg/dl)	0.98	1.8	0.4	1.28
Hepatic synthetic capacity indices	ALB (g/dl)	3.75	3.1	− 0.3	3.4
Others	PLT (*10^3^/mmc)	108.0	90.7	− 12.8	129
	AFP (ng/ml)	411.6	159.0	− 547	251.7

AST: Aspartate Transferase, ALT: Alanine Aminotransferase, GGT: Gamma-Glutamyl Transferase, ALP: Alkaline Phosphatase, PLT: platelet count (× 10^3^/mmc), ALB: albumin, Bil Tot, Bil Dir and Bil Ind: total, direct and indirect bilirubin values, respectively, AFP: alpha-fetoprotein

Mean procedural duration was 108 ± 20 min.

Technical success was obtained in all cases (15/15; 100%). Clinical success was obtained in 11 cases (73%) while in 4 cases (27%), partial response was reported. Just 1 partial response case had a rapid progression of systemic disease and comorbidities up to death; for this reason, no re-treatment was realized, whereas the other 3 partial response cases were re-treated with cTACE alone to chemoembolize the residual HCC. No major complications were noted. In one case (4.8%), the development of segmental arterial-portal fistula was detected at one-month follow-up.

The results of univariate analysis are reported in Table 3.

**Table 3:** Univariate analysis of possible risk factors for clinical failure

	Clinical success		Clinical failure		*p*
Age	66.5	11 (IQR)	66.8	17.5 (IQR)	0.258
Sex					
Male	8	53%	3	20%	0.929
Female	3	20%	1	7%	
HCC Site					
Left lobe	2	13%	2	13%	0.218
Right lobe	9	60%	2	13%	
HCC volume (cm^3^)	356.5	354.7 (IQR)	558.8	890 (IQR)	0.363
Procedure duration (minutes)	110.2	21 (IQR)	103	24.8 (IQR)	0.395
Pre*-AST	46.1	38 (IQR)	26	21 (IQR)	0.327
Post**-AST	122.7	80.5 (IQR)	248	353 (IQR)	0.308
ΔAST	67.4	68.4 (IQR)	222	363 (IQR)	**0.023**
Pre-ALT	55.7	32 (IQR)	30.5	11.5 (IQR)	0.177
Post- ALT	106.7	39.7 (IQR)	177.3	305.8 (IQR)	0.750
ΔALT	42.4	56 (IQR)	121.8	226.3 (IQR)	**0.043**
Pre-GGT	167,9	157 (IQR)	113.8	146.5 (IQR)	0.284
Post-GGT	148	34 (IQR)	363	99.5 (IQR)	0.420
ΔGGT	− 5.7	11 (IQR)	− 23.5	49 (IQR)	0.280
Pre-ALP	121.1	76 (IQR)	97	55 (IQR)	0.582
Post-ALP	104.7	32.7 (IQR)	84.5	30 (IQR)	0.711
ΔALP	− 0.1	40 (IQR)	− 12.5	25 (IQR)	0.358
Pre-Bil Dir	0.34	0.4 (IQR)	0.28	0.25 (IQR)	0.999
Post-Bil Dir	0.67	0.37 (IQR)	0.58	0.65 (IQR)	0.681
ΔBil Dir	0.37	0.47 (IQR)	0.3	0.5 (IQR)	0.222
Pre-Bil Ind	0.66	0.3 (IQR)	0.65	0.5 (IQR)	0.857
Post-Bil Ind	1.2	0.6 (IQR)	1.1	0.9 (IQR)	0.750
ΔBil Ind	0.47	0.37 (IQR)	0.45	0.5 (IQR)	0.246
Pre-Bil Tot	0.99	0.7 (IQR)	0.93	0.75 (IQR)	0.952
Post-Bil Tot	1.87	0.97 (IQR)	1.68	1.55 (IQR)	0.750
ΔBil Tot	0.88	0.58 (IQR)	0.75	0.8 (IQR)	0.174
Pre ALB	3.8	0.9 (IQR)	3.7	0.53 (IQR)	0.711
Post-ALB	3.2	0.2 (IQR)	3.7	1.05 (IQR)	0.211
ΔALB	− 0.4	0 (IQR)	− 0.3	0.98 (IQR)	0.630
Pre PLT	101.1	67 (IQR)	127	80 (IQR)	0.298
Post PLT	83.4	34 (IQR)	110.8	51.5 (IQR)	0.183
ΔPLT	− 11.7	21 (IQR)	− 15.8	30.5 (IQR)	0.582
Pre-AFP	500,6	292.1 (IQR)	115	152 (IQR)	0.689
Post-AFP	220.2	214.4 (IQR)	6.2	2 (IQR)	0.928
ΔAFP	− 699.9	698.3 (IQR)	− 164.8	274 (IQR)	0.589

Pre: pre-procedural, Post: post-procedural, Δ: value changes between pre and post-procedure, AST: Aspartate Transferase, ALT: Alanine Aminotransferase, GGT: Gamma-Glutamyl Transferase, ALP: Alkaline Phosphatase, PLT: platelet count (× 10^3^/mmc), ALB: albumin, Bil Tot, Bil Dir and Bil Ind: total, direct and indirect bilirubin values, respectively, AFP: alpha-fetoprotein levels

*pre-procedural value measured within 24 h before single-session locoregional procedure, **post-procedural value measured within 24 h after single-session locoregional procedure

*statistically significant results (*p* < 0.05)

**Fig. 4:**
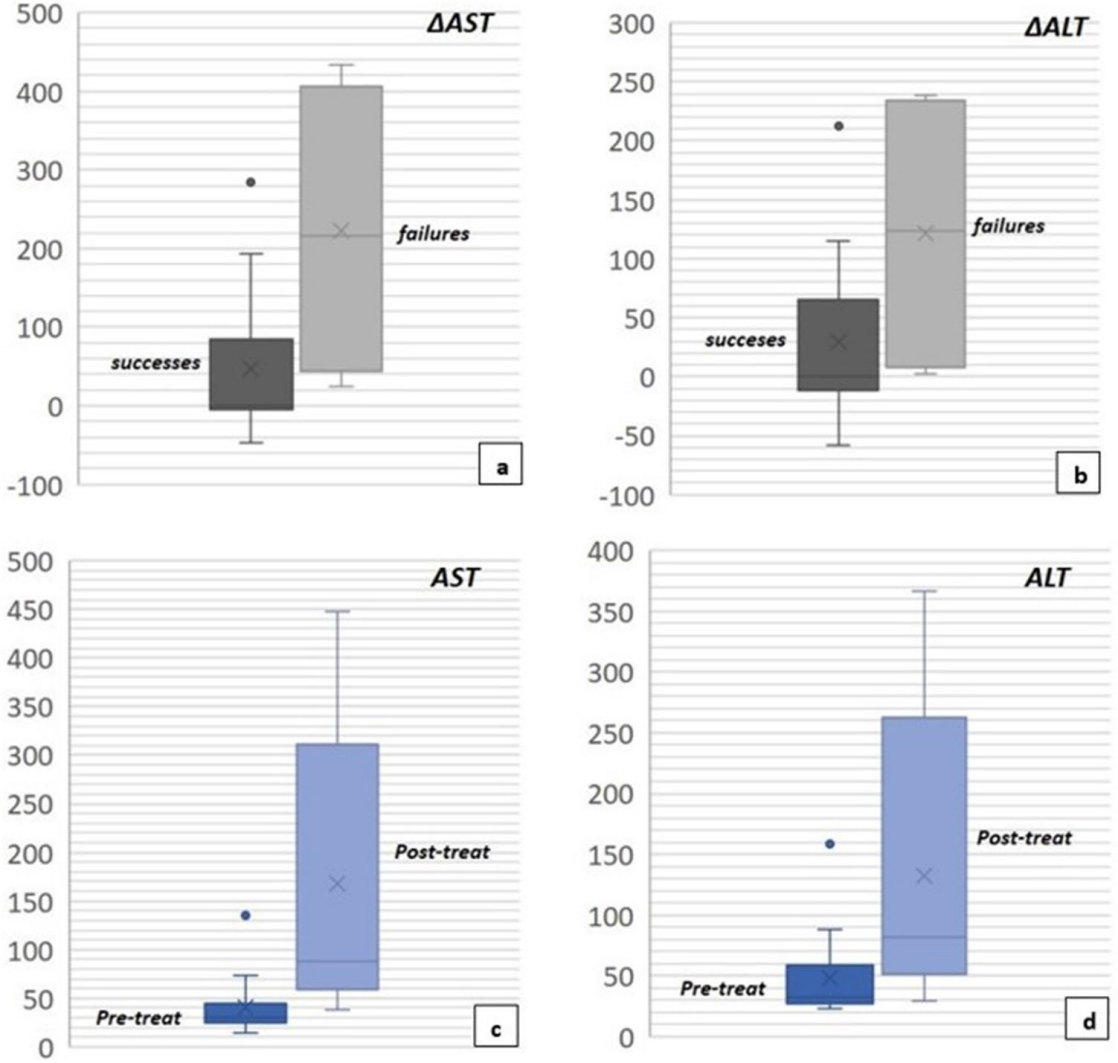
Correlation of clinical outcome with difference between pre-procedural and post-procedural AST values (ΔAST, p = 0.023, central value expressed by the median of the measurements) (**a**) and with difference between pre-procedural and post-procedural ALT values (ΔALT, p = 0.0003) (**b**). Modification of AST values between pre- and post-treatment (p = 0.00062) (**c**) and of ALT values between pre- and post-treatment (p = 0.0093) (**d**)

Statistically significant results in terms of ALT (*p* = 0.00062) and AST (*p* = 0.0093) between pre- and post-procedural values were obtained, with significantly higher ALT and AST values in post-procedural measurements (Fig. 4).

A significant difference was also observed in terms of ΔALT (*p* = 0.023) and ΔAST (*p* = 0.043) values between clinical success and clinical failure cases, with a significantly higher number of clinical failure cases registered in presence of higher ΔALT and ΔAST values (Fig. 3).

**Fig. 5:**
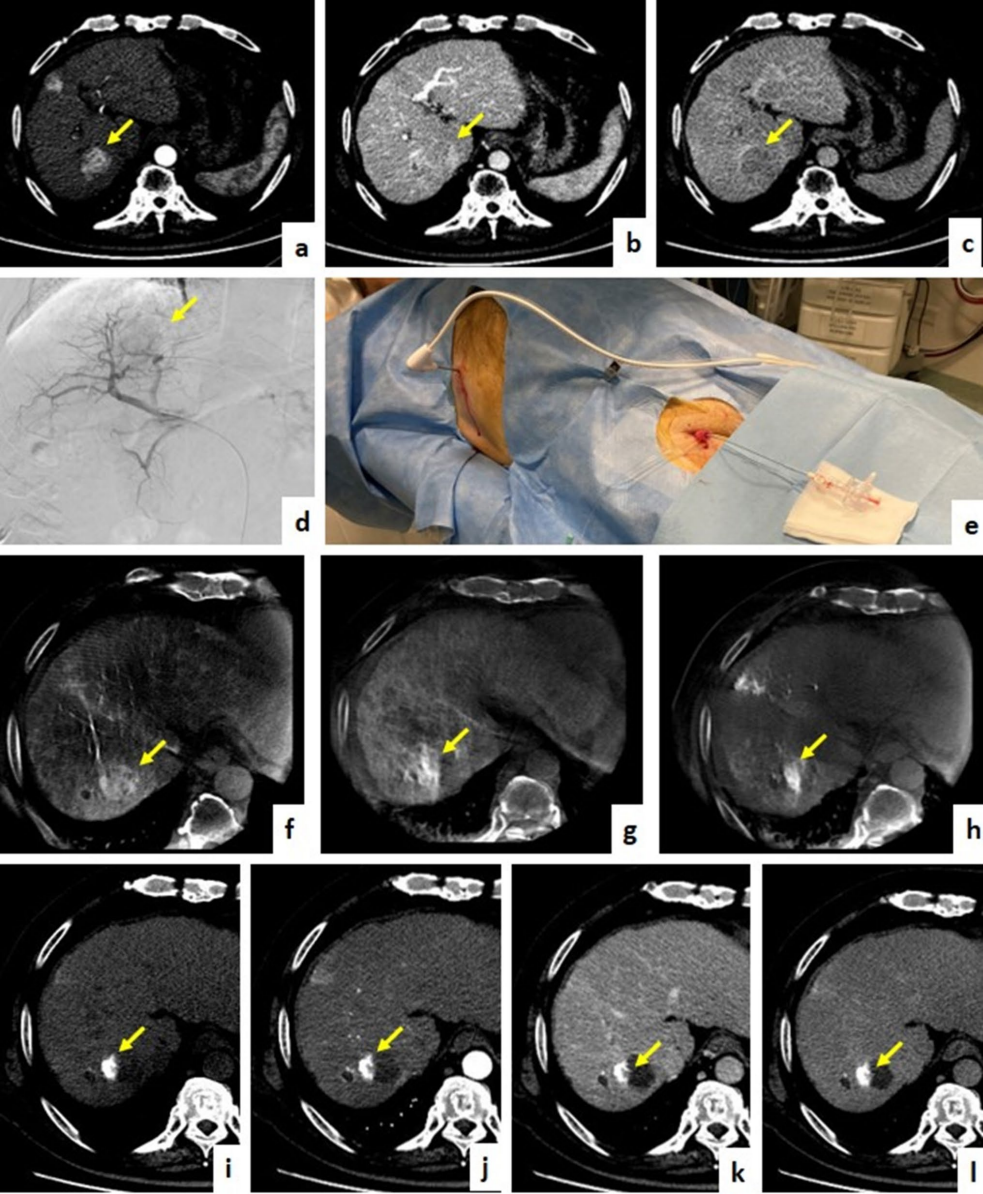
Seventy-four-years-old patient with presence of HCC lesion in the VII hepatic segment as shown in triphasic contrast enhanced CT pretreatment, arterial phase (**a**), venous phase (**b**), delayed phase (**c**). After initial angiographic study (**d**), single-session approach with MWA (**e**) followed by cTACE was performed. CBCT with intra-arterial contrast medium injection was performed before treatment (**f**) and after MWA (**g**), whereas CBCT without medium of contrast was performed after cTACE (**h**). Follow-up imaging with contrast enhanced CT shows no residual HCC and peri-lesional lipiodol distribution with central necrotic area, basal acquisition (**i**), arterial phase (**j**), venous phase (**k**), delayed phase (**l**)

A representative case of HCC lesion treated with MWA followed by cTACE in a single session, is presented in Fig. 5.

## Discussion

The combination of different types of locoregional treatments has progressively gain importance in HCC management due to its potential clinical implications. Firstly, combined treatments have demonstrated to improve local tumor control, particularly for larger tumors and for those located between early and intermediate BCLC stages [20, 26]. Additionally, combined strategies may contribute to improve patient overall survival and reduce recurrence rates [27, 28]. Although surgery and transplant remain the gold standard of HCC treatment, combined approaches are today considered an excellent option for bridging and down-staging to surgery or transplant [29].

Until today, the target of combined locoregional therapies was focused on single unresectable HCC lesions with main diameter ≥ 3 cm and classified as BCLC-A, due to the high recurrence rates after ablation as stand-alone treatment [20, 28, 30–32]. Nevertheless, several meta-analyses have obtained promising results of combined approach both in early stage and in intermediate-stage HCCs compared to single TACE or ablation [33–38].

The rationale of the combined approach arises from the possibility of overcoming the main limitations of each individual treatment through a synergistic action. The heat sink effect of ablation can be mitigated by the decreased arterial flow following TACE, thereby increasing the ablation area.

The retention of lipiodol within the nodule treated by TACE can make less challenging the evaluation of ablative margins, particularly those not clearly visible during US guidance and those at high risk of damaging the adjacent structures. TACE can also ensure the treatment of satellite nodules otherwise undetectable with ablation alone. Conversely, ablation may reduce several negative effects correlated with arterial recanalization and angiogenesis often occurring after repeated (often suboptimal) TACE procedures and commonly resulting in residual tumor and intrahepatic recurrences as well as progressive impairment of liver function [38–40]. Especially in larger tumor volumes, ablation can also reduce the expression of vascular growth factors induced by hypoxia, thus improving long-term outcomes [33, 36–38].

Despite the known theoretical advantages, precise indications regarding combined therapies are still missing in current guidelines due to several unresolved issues.

Firstly, which techniques should be preferred in combined approaches for both TACE and ablation remains controversial. Recent meta-analyses, despite the greater reported experience with radiofrequency ablation (RFA), suggest that MWA has a better tumor response and long-term survival than any other ablation technique [34, 41]. In this study, cTACE was preferred to DEB-TACE according to literature, due to many advantages such as a stronger heat conduction, the peri-portal embolization, and a better visibility of treated areas and satellite lesions [30, 42].

Treatment sequence remains another matter of debate. Despite most authors prefer TACE followed by ablation, in our series, MWA before cTACE was adopted due to known benefits such as the stimulation of necrosis on peripheral tumor areas thus exposed to higher drug concentrations, the increased vascular permeability in the surrounding ablation area due to the exposure to sublethal heating, and the higher drug concentration on a relatively smaller volume of residual viable tissue [43, 44]. In contrast to the previously hypothesized loss in efficacy of drugs injected by TACE if exposed to high temperatures, recent studies demonstrated that the sublethal hyperthermia of the periablation area enhances the anticancer activity of doxorubicin [45].

Lastly, there is a lack of relevant literature regarding the timing of combination therapy. It is known that time interval should be carefully decided in order to ensure the balance between successful tumor eradication and preservation of liver function. Most studies recommend a variable time interval from 2 to 5 weeks between ablation and TACE to allow liver function recovery, discouraging single-session approaches [46–48]. Others believe that a time interval of 0–2 days is sufficient [20].

In accordance with combined early sequential RFA + TACE results, our single-session approach demonstrated encouraging technical and clinical success rates, respectively, of 100% and 73%, without major complications [46]. Furthermore, no significant modifications between pre- and post-treatment values in most of laboratory liver function parameters were observed, except for a transitory increase in AST and ALT values. All the liver injury indices have subsequently return to normal values within one week after combined procedures, suggesting a transitory impairment rather than an acute liver injury.

An increase in AST and ALT values had been previously reported by Yuan et al., despite the fact that the more conservative sequential approach was preferred in this series [48]. Even in cases of solely TACE treatment, higher variations in AST and ALT were observed [49]. This could be explained by non-target chemoembolization and consequent higher hepatic toxicity due to a greater drug spreading within the healthy liver parenchyma [49].

Other pre-procedural laboratory values have been extensively analyzed to identify possible predictive factors of treatment failure, most of them with limited availability [27, 50, 51]. In our preliminary series, higher AST and ALT variations were significantly related with a poor clinical success, suggesting these parameters could be monitored in the first days after single-session combined treatments to predict negative outcomes.

This study has several and important limitations including the retrospective and single-center design, the small sample size, and the limited follow-up. Moreover, missing data regarding comorbidities, as well as previous locoregional and systemic treatments might have biased the local progression outcomes.

To the best of our knowledge, the present series is the first investigating the outcomes of combined treatments performed in single-session and employing exclusively MWA as ablative technology and conventional TACE as intra-arterial strategy. Furthermore, the results demonstrate that minimizing the time interval between the two different treatment modalities resulted in only a mild liver function impairment despite the maximum synergistic effect.

In conclusion, the single-session approach showed encouraging results in terms of safety, technical and clinical success with the advantage of reducing number of hospitalizations, length of stay and patient discomfort. Larger populations, multicentric and prospective controlled studies comparing single-session and sequential combined therapies are required to validate these preliminary results.

## Data Availability

The datasets generated during the current study are available from the corresponding author on reasonable request.
